# Novel insights into DNA methylation and its critical implications in diabetic vascular complications

**DOI:** 10.1042/BSR20160611

**Published:** 2017-03-15

**Authors:** Jia Zheng, Jing Cheng, Qian Zhang, Xinhua Xiao

**Affiliations:** 1 Department of Endocrinology, Key Laboratory of Endocrinology, Ministry of Health, Peking Union Medical College Hospital, Diabetes Research Center of Chinese Academy of Medical Sciences & Peking Union Medical College, Beijing 100730, China; 2 Key Laboratory of Cardiovascular Remodeling and Function Research, Chinese Ministry of Education and Chinese Ministry of Public Health, Department of Cardiology, Qilu Hospital of Shandong University, Jinan, China

**Keywords:** Epigenetics, DNA methylation, Atherosclerosis, Diabetic cardiomyopathy, Diabetic nephropathy, Diabetic retinopathy

## Abstract

Recent epidemiological and clinical studies have shown that type 2 diabetic patients can develop diabetic vascular complications even after intensive glycaemic control. It has been suggested that this phenomenon could be explained by the hypothesis of ‘metabolic memory’. The underlying mechanisms between these enduring effects and the prior hyperglycaemic state are still not well understood. Preliminary studies demonstrate that hyperglycaemia can regulate gene expression by epigenetic modifications, such as DNA methylation, which can persistently exist even after glucose normalization. Increasing evidence shows that epigenetic mechanisms may play a substantial role in the pathophysiology of diabetes and its associated vascular complications, including atherosclerosis, diabetic cardiomyopathy (DCM), nephropathy and retinopathy. In this review, we will examine the growing role of DNA methylation in diabetes and its vascular complications, thus it can provide critical implications for the early prevention of diabetes and its vascular complications.

## Introduction

### Epidemiology of diabetes and its vascular complications

The epidemic of diabetes is a serious and growing public health problem. In 2015, the International Diabetes Federation (IDF) declared that 415 million people have diabetes and the number will rise to 642 million by 2040 worldwide [1]. Diabetes is yielding enormous effects on individuals, public health and social economy. Despite prominent advances in diabetes treatment, glucose monitoring and biomarkers of glycaemic control, detrimental vascular complications still remain in most diabetic patients [2]. Diabetes is associated with significantly accelerated rates of several macrovascular complications such as atherosclerosis, diabetic cardiomyopathy (DCM) and other cardiovascular diseases and microvascular complications such as nephropathy and retinopathy [3,4]. Diabetic cardiovascular diseases are the main cause of disability and death of diabetic patients [5]. It reports that the risk of cardiovascular complications occurring on diabetic patients is two to four times higher than the healthy people [6]. In addition, it indicates that approximately 20–30% of type 2 diabetic patients have severe renal impairments, with their glomerular filtration rate lower than 60 ml/min [7]. Diabetic retinopathy is the most common microvascular complication of diabetes, and it remains a leading cause of legal blindness and visual impairment in the working-age population in the developed world [8].

### Diabetic complications and metabolic memory

**Figure 1:**
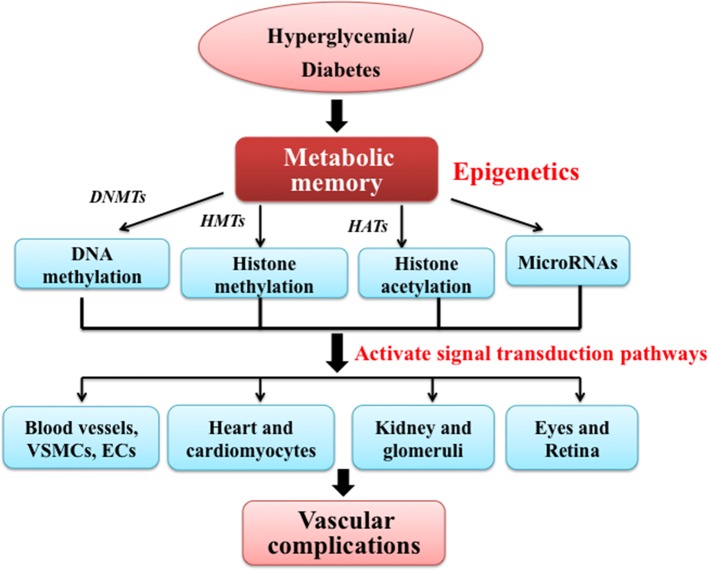
Schematic diagram shows the role of epigenetic mechanisms in metabolic memory and diabetic vascular complications Metabolic memory is the phenomenon of diabetic vascular stresses persisting after glucose normalization in diabetic patients because of early a hyperglycaemic environment. Increasing studies show that epigenetics may be the underlying mechanisms, which can explain metabolic memory. Epigenetic mechanisms including DNA methylation, histone methylation, histone acetylation, are regulated via the action of corresponding DNMTs, HMTs and HATs. In addition, miRNAs can also play a significant role in the process. Together they activate multiple signal transduction pathways and regulated related gene expression, involving blood vessels, heart, kidney and eyes. Then, it can increase the susceptibility of macrovascular complications such as atherosclerosis, DCM and microvascular complications such as nephropathy and retinopathy. ECs, endothelial cells; VSMCs, vascular smooth muscle cells.

Although the prevalence of diabetes and its vascular complications are increasing, the pathogenesis of diabetes and its complications have not been clearly understood. There is growing evidence supporting the role of ‘metabolic memory’ in diabetic complications and metabolic memory plays a critical role in the development of vascular complications in diabetic patients [9,10]. Metabolic memory is the phenomenon of diabetic vascular stresses, which can persist after glucose normalization in diabetic patients due to the early hyperglycaemic environment [11]. Hyperglycaemia appears to be remembered in organs such as the vessels, heart, kidney and eyes [11]. This metabolic memory phenomenon emerged from two large clinical trials: the Diabetes Complications and Control Trial (DCCT) [12] and its follow-up Epidemiology of Diabetes Interventions and Complications (EDIC) trials [13]. It showed that patients on the standard treatment regimen during the DCCT still had a higher incidence of microvascular diabetic complications such as nephropathy and retinopathy, compared with their counterparts receiving intensive therapy throughout the trial several years after switching to intensive therapy [13]. This suggests that early metabolic control has enduring effects in diabetes and its complications. However, little is known about the molecular mechanisms underlying metabolic memory. Increasing studies show that epigenetics may be the underlying mechanisms, which can explain metabolic memory [14]. Epigenetic processes play a critical role in regulating tissue-specific gene expression and hence alterations in these processes may induce long-term changes in gene function and metabolism, which can persist throughout the course of diseases [15]. Epigenetic mechanisms including DNA methylation, histone methylation, histone acetylation, are regulated via the action of corresponding DNA methyltransferases (DNMTs), histone methyltransferases (HMTs) and histone acetyltransferases (HATs). In addition, miRNAs can also play a significant role in the process [16]. Together, they activate multiple signal transduction pathways and regulate related gene expression, involving blood vessels, heart, kidney and eyes [17]. Therefore, it can increase the susceptibility of macrovascular complications such as atherosclerosis, DCM and microvascular complications such as nephropathy and retinopathy. A schematic diagram (see Figure 1) shows the role of epigenetic mechanisms in metabolic memory and diabetic vascular complications.

## Epigenetics and DNA methylation

### A glimpse at epigenetics

Epigenetics has been widely accepted as the heritable changes in gene function, which is independent of DNA sequence [18]. It can be inherited among generations steadily by mitosis and meiosis through cell differentiation and division [19]. Epigenetic changes are crucial for the development and differentiation of the various cell types in an organism and are often involved to switch on or off the genes that produce permanent changes associated with the differentiation of diverse cell type [20]. Epigenetic modifications consist of three basic processes, including DNA methylation, histone modification and non-coding RNAs [18]. In this review, we will examine the role of DNA methylation in diabetes and its vascular complications and recent progress that have significantly accelerated this field.

### What is DNA methylation?

**Figure 2:**
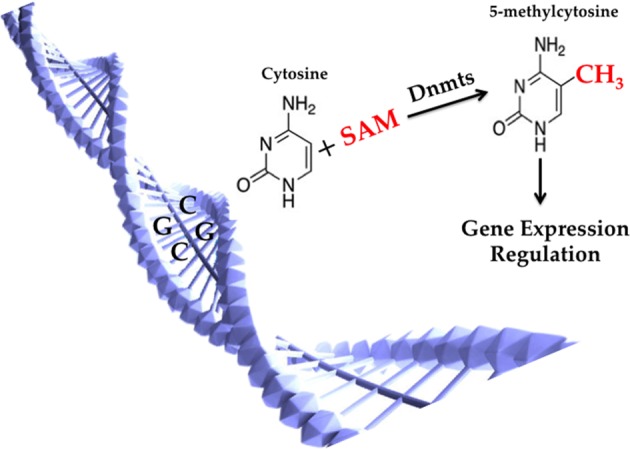
Molecular mechanisms of DNA methylation DNA methylation is exerted by DNMTs at the 5′-position of cytosine residues in CpG dinucleotides (the p denotes the intervening phosphate group) by transferring methyl groups from SAM, thus 5-methylcytosine is formed. DNA methylation of promoter CpG islands generally can regulate gene expression.

DNA methylation is the earliest discovered and most important epigenetic modification with extensive investigations. DNA methylation is exerted by DNMTs at the 5′-position of cytosine residues in CpG dinucleotides (the p denotes the intervening phosphate group) by transferring methyl groups from S-adenosyl methionine (SAM), thus 5-methylcytosine is formed [21]. Most CpG dinucleotides are often grouped in clusters at the 5′-regulatory regions of many genes, which are called CpG islands. DNA methylation of promoter CpG islands generally can regulate gene expression (Figure 2) [22]. As one of the most stable epigenetic modifications, DNA methylation is essential for normal development and is associated with a number of key processes and metabolic diseases, such as obesity [23], type 2 diabetes [24] and cardiovascular diseases [25].

## DNA methylation and diabetic vascular complications

### DNA methylation and atherosclerosis

It is well known that atherosclerotic cardiovascular disease is the major cause of morbidity and mortality in diabetes. Atherosclerosis occurs earlier and with greater severity in diabetic patients, leading to a much higher risk of limb ischaemia, myocardial infarction and stroke [26]. Arterial smooth muscle cell migration and proliferation are central features in atherogenesis. It showed that genomic hypomethylation was observed during atherogenesis in human, mouse and rabbit lesions, which correlated with increased transcriptional activity. Then, they identified that DNMT was expressed in various types of atherosclerotic lesions [27]. Furthermore, whole genome hypomethylation was present in peripheral blood mononuclear cells and aortas of 4-week-old ApoE-null mice, preceding any histological sign of atherosclerosis, which means that DNA methylation maybe a potential biomarker for early atherosclerosis in diabetic patients [28]. In VSMCs, ECs and mouse models, altered DNA methylation of several candidate genes linked with atherosclerosis was identified, including vasodilator endothelial nitric oxide synthase, hypoxia-inducible factor-1a and matrix metalloproteinases [29]. Other risk factors of cardiovascular diseases, such as hyperhomocysteinaemia, hypercholesterolaemia and inflammation have also been implicated in differential DNA methylation associated with atherosclerosis [30].

### DNA methylation and DCM

Cardiovascular complications are a primary cause of mortality and morbidity in diabetic patients. DCM was first proposed in 1972, which is a distinct primary disease process, independent of coronary artery disease [31]. It can lead to heart failure in diabetic patients, characterized by left ventricular hypertrophy and decreased diastolic compliance [32]. There is very scant information on the epigenetic regulation of the genes involved in the pathophysiology of DCM. In a streptozotocin (STZ)-induced diabetic rat model, it showed that demethylation of liver X receptor α (LXRa) was found to be responsible for its increased expression in myocardial ventricles of diabetic rats [33]. In another study, it indicated that tumour necrosis factor (TNF)-α increased DNMT levels, thus enhancing the methylation in the sarcoplasmic reticulum Ca-ATPases (SERCA2a) promoter region with a result of reducing SERCA2a expression in cardiomyocytes [34]. One previous study of type 2 DCM patients demonstrated that demethylation of the CpG islands in the Kelch-like ECH associated protein 1 (Keap1) promoter activated the expression of Keap1 protein, which then increased the targeting of nuclear factor-like 2 for proteosomal degradation [35]. Genes involved in renin–angiotensin–aldosterone system (RAAS) pathway can be up-regulated in DCM and this results in cardiac hypertrophy. Bogdarina et al. [36] showed that the proximal promoter of the AT1b angiotensin receptor gene in the adrenals was significantly undermethylated [36]. These data suggest that expression of related genes is regulated by DNA methylation and may have a significant role in the pathophysiology of DCM.

### DNA methylation and diabetic nephropathy

Diabetic nephropathy is a serious microvascular complication of diabetes mellitus and and has become the most common cause of end-stage renal disease (ESRD). There is increasing evidence to suggest that dysregulation of the epigenome is involved in diabetic nephropathy [37]. One clinical study showed that whole blood genomic DNA from type 1 diabetic patients with diabetic nephropathy exhibited differential DNA methylation patterns at 19 genes including protein unc-13 homologue B (UNC13B), relative to those without nephropathy [38]. Pirola et al. [39] found that hyperglycemia was associated with hypermethylation changes localized to regions close to transcription start sites of primary vascular cells. One recent study indicated that the average methylation ratio of the let-7a-3 promoter in the diabetic nephropathy individuals was significantly higher than that in the type 2 diabetic patients without nephropathy, which was relevant to the down-expression of let-7a-3 in diabetic nephropathy patients [40]. Furthermore, several differentially methylated genes were also identified in DNA extracted from saliva of diabetic patients with ESRD compared with patients with chronic kidney disease who did not progress to ESRD [41]. However, more extensive studies with larger samples should be conducted to comprehensively address the significance of aberrant gene methylation in diabetic nephropathy.

### DNA methylation and diabetic retinopathy

Diabetic retinopathy remains one of the major causes of blindness in adults. Clinical and experimental studies have demonstrated that hyperglycaemia has long-lasting effects on the retina and the damage continues even after termination of the hyperglycaemic abberration [42]. Epigenetic modifications may be the underlying mechanisms that can explain this phenomenon. Genome-wide analysis of DNA methylation in subjects with type 1 diabetes identified epigenetic modifications associated with proliferative diabetic retinopathy, which is a primary cause of vision loss in subjects with diabetes [43]. Matrix metalloproteinase-9 (MMP-9) has an important role in the pathogenesis of diabetic retinopathy. Recently, Kowluru et al. [44] showed that the regulation of hypomethylation of retinal MMP-9 promoter regulated its transcription and prevented mitochondrial damage in STZ-induced diabetic C57BL/6J mice. In STZ-induced diabetic Wistar rats, continued hypermethylation of the CpG sites at the regulatory region of polymerase γ 1 (POLG), which is the catalytic subunit of the mtDNA replication enzyme, affected its binding to the mtDNA, which were associated with continued progression of diabetic retinopathy [45]. This indicates that modulation of DNA methylation can regulate the progression of diabetic retinopathy.

Taken together, the aforementioned clinical studies, animal models and *in vitro* experiments have shown that DNA methylation may play a substantial role in the pathophysiology of diabetes and its associated vascular complications, including atherosclerosis, DCM, nephropathy and retinopathy. The relevant evidence was summarized in Table 1.

**Table 1:** Summary of relevant studies about DNA methylation and diabetic complications

Vascular complications	Species	Regulated genes	Methylation	Reference
Atherosclerosis	Human, mouse and rabbit	Whole genome	Hypomethylation	Hiltunen et al. [27]
Atherosclerosis	ApoE-null mice	Whole genome	Hypomethylation	Lund et al. [28]
Atherosclerosis	VSMCs, ECs and eNOS-null mice	Hypoxia-inducible factor-1a, vasodilator endothelial nitric oxide synthase and matrix metalloproteinases	Hypermethylation and hypomethylation	Matouk and Marsden [29]
Atherosclerosis	Human macrophages	Whole genome	Hypomethylation	Zaina et al. [30]
DMC	STZ-induced diabetic rat	LXRa	Demethylation	Cheng et al. [33]
DMC	HL-1 cardiomyocytes	SERCA2a	Hypermethylation	Kao et al. [34]
DMC	Type 2 diabetic patients	Keap1	Demethylation	Liu et al. [35]
DMC	Wistar rats	AT1b angiotensin receptor gene	Undermethylated	Bogdarina et al. [36]
Diabetic nephropathy	Type 1 diabetic patients	19 CpG sites	Differential DNA methylation	Bell et al. [38]
Diabetic nephropathy	Primary vascular cells	Whole genome	Hypermethylation	Pirola et al. [39]
Diabetic nephropathy	Type 2 diabetic patients	Let-7a-3	Hypermethylation	Peng et al. [40]
Diabetic nephropathy	Diabetic patients	Whole genome	Differentially methylated	Sapienza et al. [41]
Diabetic retinopathy	Type 1 diabetic patients	233 unique genes including TNF, CHI3L1	Differential DNA methylation	Agardh et al. [43]
Diabetic retinopathy	STZ-induced diabetic C57BL/6J mice	MMP-9	Hypomethylation	Kowluru et al. [44]
Diabetic retinopathy	STZ-induced diabetic Wistar rat	POLG	Hypermethylation	Tewari et al. [45]

CHI3L1, chitinase-3-like protein 1.

## Epigenetic reversibility and potential therapeutic perspectives

Traditionally, it was considered that epigenetic modifications were static in the regulation of gene expression. However, this idea is now being altered and epigenetic marks, including DNA methylation are dynamic induced by some factors. Some preliminary studies have showed that DNA methylation plays an important role in the reversibility and treatment of diabetic complications. One of the first study showed that epigenetic modifications was reversibly regulated in diabetic complications. It demonstrated that treatment of type 2 diabetic db/db mice with the angiotensin II type 1 receptor (AT1R) blocker losartan not only ameliorated diabetic nephropathy, but also reversed epigenetic changes. More specifically, it showed significantly increased expression of HMTs and HATs in db/db mice. However, these increases were abolished in losartan-treated mice, accompanied with decreased blood pressure, mesangial hypertrophy and proteinuria [46]. Lou et al. [47] aimed to investigate the effects of resveratrol (trans-3,5,40-trihydroxystilbene) on the expression of pro-inflammatory cytokines such as IL-1β, IL-6, TNF-α and IFN-γ in diabetic rat aortas and the potential epigenetic mechanisms involved. It showed that the expression levels of pro-inflammatory cytokines were significantly lower in the resveratrol-treated diabetic group. Furthermore, the untreated group showed reduced levels of DNA methylation at the specific cytosine phosphate guanosine sites of IL-1β, IL-6, TNF-α and IFN-γ and these levels were reversed by resveratrol [47]. Thus, although the studies were limited, they implicated that epigenetic modifications may be one of the protective mechanisms and it can be reversible, which may be used as a therapeutic tool targeting diabetic vascular complications.

## Conclusions

In summary, DNA methylation plays a critical role in the pathogenesis of diabetic complications. A better understanding of the role and mechanism of DNA methylation and diabetic complications can inspire critical implications for the early prevention of type 2 diabetes and provide unique opportunities to develop novel therapeutic approaches of diabetic complications. Furthermore, increasing evidence shows that epigenetic modifications are not static, which are dynamic and even reversible. Therefore, in view of the reversibility of epigenetic mechanisms, intervention with pharmaceuticals or other interventions during early course of diabetes may ameliorate its complications in later life, which can generates long-lasting effects.

## Abbreviations

ApoE-null: apolipoprotein E-null
AT1b: angiotensin II receptor, type 1b
DCCT: diabetes complications and control trial
DCM: diabetic cardiomyopathy
DNMT: DNA methyltransferase
EC: endothelial cell
eNOS: nitric oxide synthase 3, endothelial cell
ESRD: end-stage renal disease
HAT: histone acetyltransferase
HMT: histone methyltransferase
IFN-γ: interferon-γ
IL-1β: interleukin-1β
Keap1: Kelch-like ECH associated protein 1
LXRa: liver X receptor α
MMP-9: matrix metalloproteinase-9
POLG: polymerase γ 1
SAM: S-adenosyl methionine
SERCA2a: sarcoplasmic reticulum Ca-ATPases
STZ: streptozotocin
TNF: tumour necrosis factor
VSMC: vascular smooth muscle cell
